# Filamentous cyanobacteria growth assessment using fluorinated ethylene propylene microcapillaries

**DOI:** 10.1557/s43577-024-00813-7

**Published:** 2024-11-19

**Authors:** David M. S. Silva, Raquel Amaral, Nuno M. Reis, Paulo R. F. Rocha

**Affiliations:** 1 Bioelectronics & Bioenergy Research Lab, Centre for Functional Ecology-Science for People & the Planet, Associate Laboratory TERRA, Department of Life Sciences, University of Coimbra, Coimbra, Portugal; 2 Department of Chemical Engineering and Centre for Bioengineering and Biomedical Technologies, University of Bath, Bath, Claverton Down, UK

**Keywords:** Filamentous cyanobacteria, Microcapillary, Rate of increase of population, Algal bloom, *Oscillatoria*

## Abstract

**Abstract:**

Filamentous cyanobacteria originate toxic harmful algal blooms (HABs) in aquatic ecosystems, severely impacting freshwater ecosystems and life. Despite being natural bloomers, these microorganisms are challenging to handle *in vitro*, due to the formation of aggregates with entangled filaments. Consequently, their precise growth dynamics, although vital to timely predict HABs, remains inaccessible. Here, we precisely assessed growth of the HAB forming cyanobacteria *Oscillatoria nigroviridis*, by cultivating filament suspensions in transparent, gas permeable, and portable fluoropolymer microcapillary strips. Direct optical observation of *O. nigroviridis* growth revealed shorter filaments comprising less than 50 cells grew at a slower rate, *dN/dt* = 0.09 cell/day compared to filaments comprising more than 50 cells, with *dN/dt* up to 0.47 cell/day. The fourfold increase in *dN/dt* is suggested as part of the blooming strategy of the microorganism. This work suggests that fluoropolymer microcapillary strips can be used for effortless sampling and high-resolution monitoring of HABs.

**Impact statement:**

Climate change is increasing the occurrence of episodes of harmful algal bloom, where uncontrolled growth of noxious cyanobacteria such as* Oscillatoria* species has detrimental outcomes in both the environment and biomass production industry, consequently, impairing human and animal health due to the production of toxic or bioactive compounds. In particular, the study of growth dynamics of* Oscillatoria* species has been limited to unprecise methods due to complications with aliquoting filamentous biomass. Fluoropolymer microcapillary strips provide an ideal miniaturized platform for sampling, cultivation, and growth monitoring of *O. nigroviridis* strain UHCC 0327, which paves the way to foster better water quality management tools.

**Graphical abstract:**

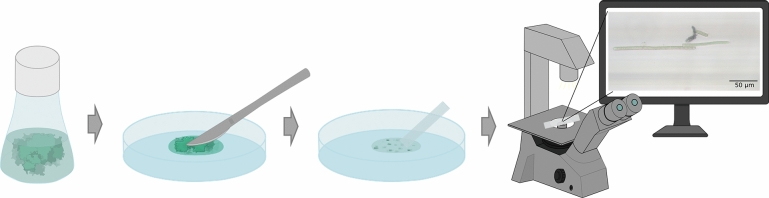

**Supplementary information:**

The online version contains supplementary material available at 10.1557/s43577-024-00813-7.

## Introduction

Cyanobacteria are the oldest oxygenic phototrophs (~2.5 billion years) with an array of physiological, morphological, and ecological adaptations in response to stressors, including geochemical, climate change, and nutrient deficiencies and alterations.^1^ This group of photosynthetic bacteria exhibits a diverse variety of species scattered throughout different environments, including freshwater, marine, hypersaline, hot springs, and terrestrial habitats.^2^ They contribute to carbon fixation and many filamentous genera are capable of fixing nitrogen.^3,4^ Due to global warming and other human-derived nutrient-rich discharge and increase in water temperatures, anomalous proliferation of cyanobacteria and other bloom-forming phytoplankton often leads to the formation of uncontrolled harmful algal blooms (HABs). These events cause eutrophication and the deoxygenation of bottom waters, with a dramatic toll on the affected food web and ecosystems.^5,6^ In freshwater ecosystems, cyanobacteria are the main bloomers, including many toxic subsurface species of *Oscillatoria*.^1^ Due to the presence of collapsible gas vacuoles, blooming^1^ filamentous cyanobacteria such as *Oscillatoria* are able to migrate between the radiant-rich surface to the nutrient-rich bottom.^1,7^ Certain species are known to produce toxins that cause an unpleasant earthy/moldy taste and odor in water sources such as geosmin^8^ and methylisoborneol.^9^ Additionally, they can also produce toxins such as anatoxin-a^10,11^ that contaminate aquatic environments, harmful to both animals and human beings.^1^

Despite the relevance of filamentous cyanobacteria, their *in vitro* study has been limited by a demanding maintenance, requiring frequent nutrient refreshing to prevent culture loss and lack of growth assessment methodologies with effective technical solutions to overcome. Conventional methodologies used to study the kinetics of growth of photosynthetic microorganisms are generally not applicable for filamentous species. A common method for growth assessments is manual or automated cell counting of an aliquot of the culture over a period of time. Conventional cell counting methods have been conveniently applied for growth estimations of unicellular organisms suspended in liquid medium. Yet, cell counting methods are not suitable to assess growth in colonial morphologies even with very diluted samples.^12^

With complex species such as those with colonial morphologies, estimation of growth is performed by employing indirect methods such as weighing biomass and estimating its increase over a period of time.^13,14^ *Oscillatoria* species are filamentous and therefore, do not grow in typical numerical exponential progression over time as unicellular species do. Filaments elongate by adding cells, increasing the length of the trichome (chain of cells sometimes referred to as filament) by breakage, which creates shorter filaments. Shorter filaments are lighter than longer filaments and more prone to be transported by water currents and colonize new areas in surface water.^15^ They are similar to parental trichomes, which distinguish them from hormogonia of other filamentous genera.^16^ Size of filaments has been related with reproduction^15^ and adaptations to nutrient scarcity in *Oscillatoria simplicissima*, which produces shorter filaments and hormogonia, for improving the surface area-to-volume ratio. This is a highly efficient strategy to withstand low availability of phosphorus and nitrogen (P and N).^15^ Similar to other filamentous species, *Oscillatoria* filaments often grow and tend to entangle, which is an important feature in natural settings; however, when current *in vitro* methods are employed, this impairs the pipetting of aliquots and creates inconsistency in the growth-related determinations, especially those based on biomass increase weighing over time, due to the presence of a spectrum of sizes of filaments in initial population.^17–19^

Chlorophyll-a extraction has also been used to measure culture growth in *Oscillatoria*,^15,20–22^ although with a significant variability regarding culturing methods and duration. As chlorophyll content may vary not only with cell number, but also with the cell metabolic state,^23^ chlorophyll-based growth measurements are highly biased indirect methods that could result in imprecise estimations. Different species may have different metabolic contents, which also adds another variability layer to the use of metabolic-based growth estimation methods.^24^ Additionally, *Oscillatoria* forms filament aggregates *in vitro* and consequently, no study can be found in literature providing a detailed description of how aliquots were taken from starting biological material, regardless of the estimation methodology.

In this work, we developed a direct, high-resolution method to monitor the growth of filamentous HAB-forming species *Oscillatoria nigroviridis*,^25^ specifically the strain *O. nigroviridis* UHCC 0327, by cultivating suspensions of filaments in transparent, gas-permeable, portable microcapillary strips^26^ mass-manufactured from fluorinated ethylene propylene (FEP) copolymer. Direct optical observation and image analysis were used to track growth curves of *O. nigroviridis* over four weeks. Cell increments in the filaments were directly monitored noninvasively through the transparent microcapillaries, an unprecedented methodology employed to filamentous photoautotrophs. We established a relation between longer filaments, which contributes to fast-growing “mats” of *O. nigroviridis*, comprising more than 100 stacked cells with higher elongation rate and hypothesized that smaller filaments, below 50 stacked cells, are likely perceived by the organism as colonizing hormogonia, in accordance with morphological descriptions of reproduction in species of *Oscillatoria*.^16^ Furthermore, we demonstrated the long-term cultivation capability of the microcapillary strips device with a yearlong living culture without nutrient renewal.

## Materials and methods

### Monitoring device, cultivation, and loading of *O. nigroviridis*

**Figure 1:**
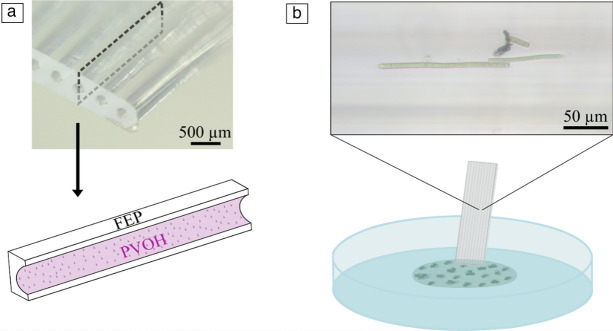
Loading and measurement setup. (a) View of the microcapillaries in the strips, with illustration of the thin layer of PVOH used for internal functionalization. (b) Loading process of the *O*. *nigroviridis* filaments suspension into the strip device. The inset shows a micrograph with *O*. *nigroviridis* filaments inside one microcapillary.

Each device consisted of a 25-mm-long fluorinated ethylene propylene (Teflon FEP) microcapillary strip, composed by 10 parallel microcapillaries with a mean diameter of 206 ± 12.6 µm (**Figure** **1**a). Although a range of diameters are available,^27^ between 100 and 500 μm, we have identified 206 μm giving the best compromise between volume interrogated, miniaturization, and capillary forces. Larger diameters would produce lower capillary rises, whereas smaller diameters would fail to load a significant number of cells per capillary. In addition, in previous studies using spherical-shaped photosynthetic microalgae,^33^ we showed that growth dynamics inside the microcapillary strips are optimum due to an effective exposure to light and gas permeability—enabled by the FEP Teflon polymer and tubular shape. The material is transparent and gas-permeable due to the nature of FEP Teflon polymer.^26^ The inner surface of the microcapillaries was previously hydrophilized by coating with poly(vinyl alcohol) (PVOH) (Figure 1a) according to Reference 26.

A culture of *Oscillatoria *was sourced from HAMBI culture collection (helsinky.fi/hambi), University of Helsinki, with the collection number UHCC 0327, and cultivated for one week in a 250-mL Erlenmeyer flask with BG11 culture medium (Sigma-Aldrich) adjusted to pH 8.2. The UHCC 0327 strain selected for our study was based on the indication on its collection file that it is a producer of taste/odor molecules geosmin and 2-methylisoborneol.^28^ The strain was identified in the collection record file as *Oscillatoria* sp. and recently identified to the species level based on 16SrRNA gene sequence with 95.27% as *O. nigroviridis*,^9^ a species that lives in habitats of a range of salinities. In this study, we adopted the species name for this strain, which follows the latest taxonomic identification available. The cultures were incubated in a growth chamber (Aralab, FC S600PLH) with a temperature of 18°C, photoperiod of 12-h:12-h light:dark, and a light intensity of 30 μmol/m^2^/s provided by cool white daylight fluorescent lamps. A portion of* O*. *nigroviridis* was transferred to a sterile Petri dish, cut with a scalpel to disassemble the filaments, and dispersed by pipetting and vortexing to create a dense and approximately homogeneous filament suspension (Figure 1b). A photograph of the filament aggregates formed in the starting Erlenmeyer culture may be seen in Online resource 1 (Supplementary information).

The suspension was then loaded into the hydrophilic microcapillary strips by positioning the strip with the open capillaries touching the filament suspension, which immediately moved into the strip by capillary action (Figure 1b). Once fully loaded, the end of strips were melted with a heated scalpel blade and sealed with a lubricating grease (75,003,786, Thermo Scientific) to ensure capillaries were fully sealed. The strips (*n* = 8) were taped to a Petri dish to enable a fixed position for image analysis procedures. The strips were then loaded with *O*. *nigroviridis* and cultivated in the growth chamber during 28 days.

### Image analysis and growth estimations

Each sealed microcapillary strip was observed under the inverted microscope (Zeiss Vert.A1), in each strip, and an area with filaments was selected for study. All filaments in the selected channels were photographed (Axiocam 208 color, Image software ZEN 3.3 Blue Edition, Zeiss) (Figure 1b) and the position of the study area was recorded on the device and the microscope stage to enable the correct position for detecting and measuring the same filaments every seven days, during four weeks. The resulting microphotographs of filaments inside the microcapillary strips were analyzed (*n* = 28) by manually selecting their borders and creating a contrast using the threshold option (Fiji 2.14.0), according to previously developed methods.^33^

**Figure 2:**
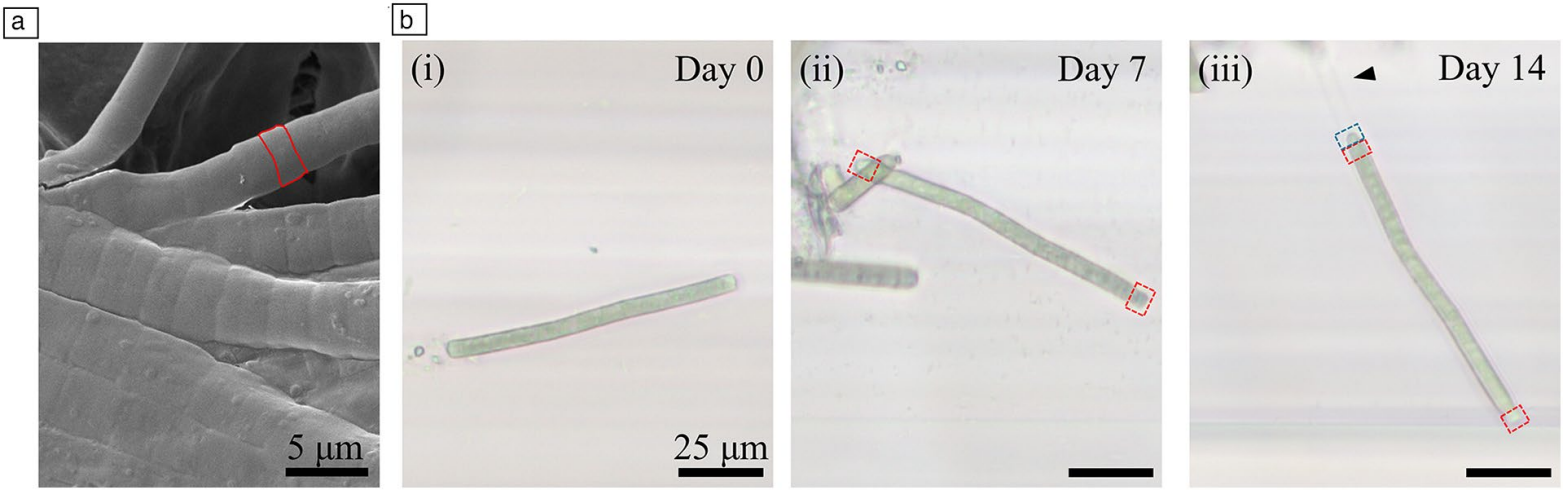
*O*. *nigroviridis* UHCC 0327 observation and determination of the area of each cell in a filament and the elongation of filaments by adding new cells apically. (a) Scanning electron microscopy micrograph showing filaments with well-defined individual cells (red highlight) (×5000). (b) One filament observed weekly, showing (i) the flat tips at day zero, corresponding to middle cells exposed after cutting and loading into the microcapillaries; (ii) filament growth after one week, elongated by the addition of two new cells with rounded apex, at the tips of the filament (highlighted in red); (iii) after two weeks, one new cell with rounded apex (highlighted in blue) formed on top of a previously added cell, which became flattened.

A sample of *O*. *nigroviridis* was also imaged using a scanning electron microscope (SEM) to measure the cells composing the filaments and to determine length and width of filaments (**Figure** **2**a). The sample was prepared by depositing a 10-nm-thick gold/palladium metallization layer, in a plasma generator Quorum SC7620 Mini Sputter Coater with a Glow Discharge System with an Au/Pd sputter target. The microstructural analysis of the surfaces was performed using a TESCAN VEGA 3 SBH Easy Probe SEM with a tungsten-heated cathode. The images were acquired with a working voltage of 5 kV and using the secondary electrons detector.

Based on SEM micrographs, we measured the mean size of each cell (*n* = 56), determined to be 1.8 ± 0.4 µm length and 3.9 ± 0.7 µm width. These measurements allowed the determination of the mean area of one cell (*A*_*cell*_). For weekly determinations of growth, we used image analysis to determine the area occupied by each filament present in the study area of the micrograph (*A*_*filament*_) and determined the number of cells of each filament by dividing its area by the mean area of one cell. The mean area of one cell was determined to be 6.844 ± 2.194 µm^2^, a value obtained by multiplying the width and length of the projected area of the cell, with a rectangular shape. The number of cells in each filament over time (*n*_*cells*_) was then calculated as follows:

1 $${n}_{cells}(t)=\frac{{A}_{filament}(t) }{{A}_{ cell}},$$

where *A*_*filament*_ represents the area obtained of a filament at time *t*, *A*_*cell*_ is the average area of a cell obtained with the SEM photographs. We obtained the filament length (*L*_*filament*_) by multiplying the number of cells of the filament (*n*_*cells*_) at a given time, by the average length of one cell (*L*_*cell*_), determined to be 1.8 ± 0.4 µm, according to

2 $${L}_{filament}(t)= {n}_{cells}(t)\times {L}_{cells}.$$

### Statistical analysis

Variance analysis was performed with ANOVA single factor and t-student two-tail test with 95% confidence, with MATLAB R2024a.

## Results and discussion

### Growth of *O. nigroviridis* UHCC 0327 occurs through elongation of the filaments by cell increments at their tips

We observed and microphotographed *O*. *nigroviridis* UHCC 0327 in the microcapillary strips over the course of four weeks, which enabled the determination of growth events with a single-cell resolution. We observed several morphological characteristics typical of *Oscillatoria* species, namely, the straight cylindrical shape of filaments, each with its own sheath. The sheath sometimes was visible at the top, when devoid of cells (Figure 2, (iii) arrowhead). Each filament was composed of disk-shaped overlapping cells, separated from one another by shallow constrictions that were recognizable as cross walls visible with SEM analysis of filaments (Figure 2a). Nevertheless, the gliding and rotation movement, characteristic of *Oscillatoria* species,^16^ previously observed in agarized medium,^29^ was not seen in the microcapillaries, where the filaments were relatively static, with slight oscillation of a loose end of a filament rarely occurring. These observations evidence that even with motile filamentous species, growth monitoring is possible, due to the liquid containment, the filaments do not have a surface to glide on.

Different size lengths of filaments were detected in the microcapillaries that were easily microphotographed. Their identification for obtaining the sequential micrographs over time was possible by combining a correct position of the image analysis setup with the observation and comparison of size and relative position with other filaments. Growth in the microcapillaries was achieved through elongation of the existing filaments, by cell division and addition of new cells at one or both apical parts of the filaments (tips) (Figure 2b (ii–iii)) with a round shape, contrary to the flat apical cells found in the fragments at day zero (Figure 2b (i)), which are middle cells, exposed after the filament suspension preparation prior to loading.

### Longer filaments exhibited higher rate of increase of population

**Figure 3:**
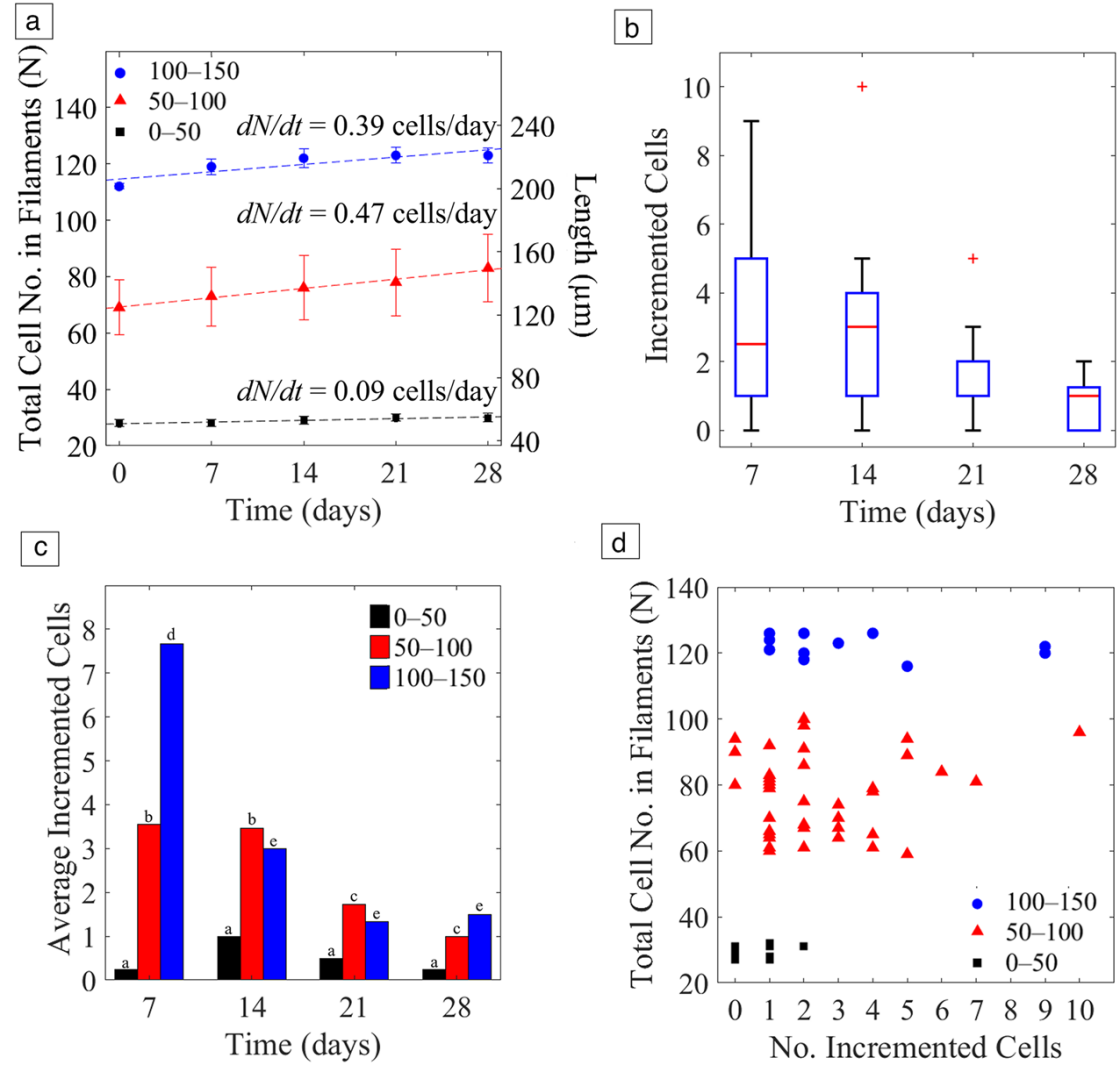
Growth of 20 *O. nigroviridis* filaments over time, in three groups according to initial size of 0–50, 50–100, or 100–150 cells. (a) Average cell number in each filament size group as a function of time fitted with a linear model, showing the rate of increase of population (slopes). (b) Box plot representation of newly formed cells (cell increment number) in measured filaments of all sizes, over 28 days. (c) Average increment each week, by filament size group, showing contribution of each group to the weekly cell increments. (d) Graphical representation of all studied filaments showing the total number of cells at start as a function of number of incremented cells. Filament groups are marked with different colors. (a–e) Denote significantly different values (*p* < 0.05), analysis of variance performed with ANOVA, and two-tailed t-student, in MATLAB R2024a.

The direct measurement of the filaments’ length from the micrographs enabled determining weekly rates of increase of population based on 20 filaments of *O*. *nigroviridis* UHCC 0327 tracked over 28 days. We established three groups of filaments based on their cell number at start, to help understand any relationship between the initial cell number and elongation of the filaments over time. The groups consisted of shorter (0–50 cells), medium size (50–100 cells), and longer filaments (100–150 cells). Increase of the total cell number in the filaments as a function of time is shown for the three groups in** Figure** **3**a.

The data were modeled with a linear fitting using MATLAB R2024a, which retrieved the slope, corresponding to the rate of increase of population, *dN/dt*. Values of *dN/dt* showed that shorter filaments (i.e., below 50 cells) grew slower, *dN/dt* = 0.09 cell/day, compared to medium and longer filaments, which grew faster, at around 0.40 cell/day (Figure 3a). The estimated *dN/dt* values correspond to real-time growth and is in full alignment with the reports based on chlorophyll estimations of *Oscillatoria* with growth rates of µ = 0.53 cell/day^23^ and µ = 0.60 cell/day,^12^ which are very likely overestimations due to the biomass-based applied methodology. There is a natural biological variability between the parallel microcapillaries, as some of those are intrinsically linked to Poisson distribution and size-dependent rate of increase of population.

This decrease in cell incrementation is likely the reason why the rate of increase of population is not higher after some weeks, since a slower cell increase also slowed down the general growth.

A general trend observed in all studied filaments, regardless of size, was the fact growth through elongation was achieved with more cells being added during the first week, and progressively less every passing week until a median of one cell added in each filament after four weeks, as seen in the box plot in Figure 3b.

This trend was more noticeable in longer filaments, which achieved an average of 7.7 ± 1.9 cells incremented after the first week, which was higher compared to 3.5 ± 1.9 cells in medium size and 0.3 ± 0.4 cells in smaller filaments, respectively, observed for the same period (**Table** **I** and Figure 3b). Shorter filaments (0–50 cells) showed unnoticeable elongation over time, on average with less than one cell incremented over the period of four weeks (Table I).

**Table I:** Average number of *O. nigroviridis* cells weekly added to the filaments, for 28 days, determined for groups of filaments according to their initial cell number. 0–50—shorter filaments initially with less than 50 cells; 50–100—medium size filaments initially with 50–100 cells; 100–150—longer filaments with 100—150 cells.

Filament Groups	Average Incremented Cells (No.)			
	Day 7	Day 14	Day 21	Day 28
0–50	0.3 ± 0.4	1 ± 0.7	0.5 ± 0.5	0.3 ± 0.4
50–100	3.5 ± 1.9	3.5 ± 2.5	1.7 ± 1.2	1 ± 0.8
100–150	7.7 ± 1.9	3 ± 0.8	1.3 ± 0.5	1.5 ± 0.5

Figure 3c made it possible to identify which groups contribute to the observed trend, where the medium size (50–100 cells) and longer filaments (100–150 cells) stand out as the main contributors to averaged elongation for all weekly measurements (*p* < 0.05). It is also noticeable that for longer filaments, the number of cells added (average incremented cells) decreases over time, after one week in filament group with 100–150 cells (*p* < 0.05) and after two weeks in medium size filaments (*p* < 0.05). A possible explanation would be that it is due to nutrient consumption, especially nitrogen, as reported in other studies for *Oscillatoria*.^15^

A combined graphical view of the three groups of filaments considering their total cell number as a function of the number of incremented cells (Figure 3d) confirmed shorter filaments incremented a maximum of two cells, whereas filaments with more than 100 stacked cells could add up to 10.

As expected, the rate of increase of population is dependent on the population size *N*. When normalizing the rate of increase of population to the size of population, *dN/dt*, to the size of population *N*, we conclude the rate of increase of population is maximum for the intermediate filaments between 50 and 100 cells.

Being a blooming organism with no specialized reproductive cells, *Oscillatoria* species reproduces vegetatively through filament breakage. Shorter, lighter fragments are able to float and move with surface water or carried by swimming animals to distant locations, where they can start another population of filaments. We observed that the longer the filament, the more they invest energy in growth, by incrementing cells and elongating further, and this observation is in accordance with other studies, where the authors argue that *Oscillatoria* withstands adverse conditions and promotes reproduction by refraining from growth and maintaining a higher number of smaller filaments.^15^ This suggests that the rate of increase of population is dependent on size of population, which creates progressively more biomass and ultimately triggers the blooming event. Because cell increments occur at the tips of filaments, we would expect equal elongation rates in all tested filament sizes, which all contain only two tips available to add new cells. However, shorter filaments grew at a much slower rate and incremented fewer cells than medium size and longer filaments (Figure 3a). This observation is in accordance with a reproductive strategy consisting of shorter filaments being more able to reach a colonizable location prior to the start of a new population. Based on evidence that *Oscillatoria* species such as *Oscillatoria brevis* produce the unpleasant taste/odor molecule geosmin at higher concentrations at the beginning of growth,^30^ we hypothesize that shorter filaments correspond to the vulnerable hormogonia seen in the natural habitats, which are isolated from the parental population and drift at the water surface. They delay growth and invest energy in the production and dispersion of such molecules as a protection strategy against grazing during the colonization process. To determine a potential relationship between shorter filaments in our study with the naturally occurring hormogonia at environmental sites, we compared the size of filaments in terms of length. The shorter filaments included in the shortest population (0–50 cells) showed an average cell number of 27.7 ± 1.3, corresponding to a length of 50 µm (**Table** **II**), which is 5× longer than the reported length of *Oscillatoria* hormogonia, which is said to be around 10 µm.^15^ Small filaments consisting of six or seven cells (~10 µm) were observed in the microcapillaries, with no cell increment visible, which indicates containment in microenvironment triggers hormogonia and lack of investment in growth. Considering a threshold length (or cell number) in the filament perceived by the organism as the minimum above which it may start growth, based on our results we situate it between six cells (10 µm) and 28 cells (50 µm).

**Table II:** Average cell number and corresponding filament length measured weekly for each size group.

Filament Groups	Day 0		Day 7		Day 14		Day 21		Day 28	
(Cell No. Range)	Average Cell No.	Length (µm)	Average Cell No.	Length (µm)	Average Cell No.	Length (µm)	Average Cell No.	Length (µm)	Average Cell No.	Length (µm)
0–50	27.7 ± 1.3	50.0	28.0 ± 1.2	50.5	29.0 ± 1.3	52.1	29.6 ± 1.3	53.2	29.95 ± 1.4	53.9
50–100	69.3 ± 9.7	124.7	72.7 ± 10.4	130.9	76.2 ± 11.4	137.2	77.8 ± 11.8	140.1	83.1 ± 12.0	149.6
100–150	111.6 ± 1.0	200.9	119.3 ± 2.7	214.8	122.2 ± 3.4	220.0	123.4 ± 2.8	222.1	123.2 ± 2.7	221.8

The microcapillaries enable further detailed study of isolated growth condition variations such as nutrient variations, presence of noxious compounds that could impair or cause excessive growth, or infinite combinations of conditions. More interestingly, these variations could be studied with the resolution of a cell (i.e., the effect could be observed and measured through image analysis) in the cells of each filament.

Microfluidic systems have been used with miniaturized cultivation chambers and channels offering full growth conditions for all applications with microalgae and cyanobacteria species.^31,32^ The rationale used for choosing this material for our study was its simplicity, portability, and previous observations of its good performance with microalgal cell suspensions. Specifically, upon testing with microalgal species *Parachlorella kessleri*, we previously observed high growth rates in the microcapillaries, with similar results as cultures in traditional batch Erlenmeyers sparged with filtered air. This was attributed to the permeability of the material to light, therefore enabling effective photosynthesis.^33^ Moreover, in previous studies, we could also determine that *Oscillatoria* sp. UHCC 0332 was able to live in confined pores of ~200 µm diameter, which was a strong indicator that the microcapillaries provided enough space.^29^

### Long-term cultivation of *Oscillatoria* during one year in the microcapillaries

**Figure 4:**
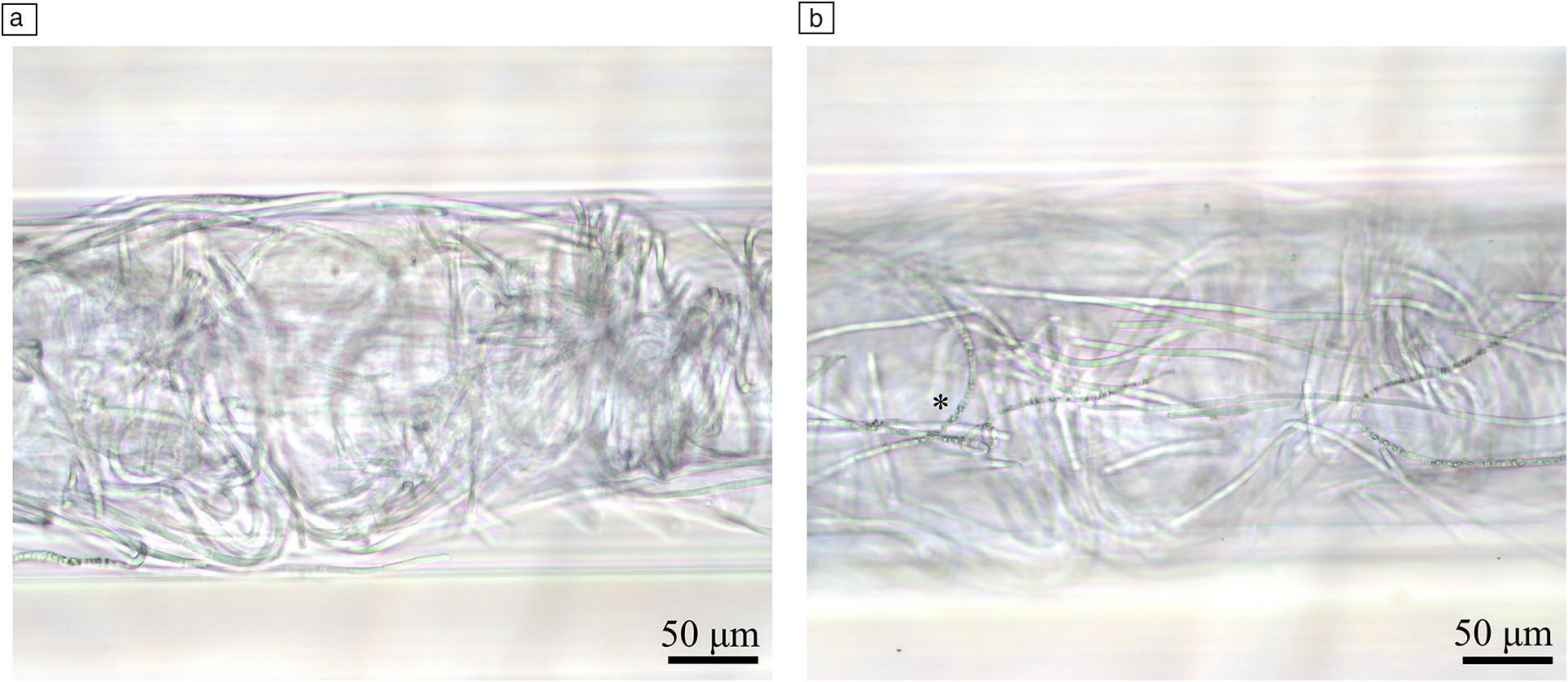
*O. nigroviridis* UHCC 0327 filaments one year after loading into microcapillary strip, close-up view of one microcapillary with living filaments, (a) filaments occupying all existing space to the capillary limits and (b) a middle section where granular cells are evidenced (*).

Some microcapillary strips were left to grow beyond the end of the experiments and were photographed one year after loading. We observed that most microcapillaries were clogged by a large quantity of filaments (**Figure** **4**), many of which were still alive. Some cells showed granules, which may correspond to reserve granules, known in *Oscillatoria* in the form of cyanophycin granules to store nitrogen as proteinaceous material and polyphosphate granules for storing phosphorus.^15^ The light and gas permeability of our FEP microcapillary device was therefore suitable for growth of photoautotrophs such as *O. nigroviridis* with potentially no light or gas limitation over time.

There is extensive work, including our own research, showing microalgae growth with similar growth rates as in standard cultivation systems. In particular, we have shown the growth of microalgae in the capillaries is sustained for 21 days, with an exponential growth rate happening earlier in the microcapillaries compared to conventional Erlenmeyers. This suggests the growth dynamics could be extrapolated. Yet, this remains unexplored and is currently under investigation.

It is well established that tracking the growth of filamentous cyanobacteria is challenging due to their oscillatory movement and entanglement of filaments—which means random multidirectional 3D growth of filaments. This has been attempted before with fluorescent probes; however, these are merely qualitative measurements rather than quantitative. Our method therefore enables direct, unlabeled optical tracking of cyanobacterial growth. We believe this is intrinsically linked to the microenvironment, which favors 2D growth aligned with the axial distance of the microcapillaries due to availability of nutrients and light. The method is yet to be demonstrated for other species of cyanobacteria and microalgae.

## Conclusions

Fluoropolymer microcapillary strips provided ideal miniaturized cultivation conditions and *in vivo* growth monitoring of a selected strain of the HAB-forming cyanobacteria genus *Oscillatoria*, due to its light and gas permeability, two essential aspects of photosynthesis. Growth occurred not by the formation of new filaments but by cell division at the tips of each existing filament, causing its elongation in length. Longer filament size groups having 50–100 and 100–150 cells, elongated more than the shorter filament group (below 50 cells), with cell increments more evident in the first two weeks after starting of the cultivation in the microcapillaries. We found the rate of increase of population was dependent on population size, with filaments of medium size (50–100 cells) and longer (100–150 cells) growing 4× faster compared to shorter filaments (50 cells or less). This growth behavior is in alignment with the characteristics of *Oscillatoria* species, viz., the longer the filaments, the more likely they are to form significant aggregates and thrive. Shorter filaments of the approximate size of hormogonia showed very slow growth, due to their reproductive mission of floating and colonizing away. Growth of HAB-forming *O. nigroviridis* may be monitored with great precision due to the employment of a real-time monitoring microcapillary device, which could have great impact for water management decision-making and future prevention of HAB-related catastrophes.

## Supplementary information

Below is the link to the electronic supplementary material.

Supplementary file1 (PDF 169 KB)

## Data Availability

The authors will make data available upon request.
