# Supplementary material for: Filamentous cyanobacteria growth assessment using fluorinated ethylene propylene microcapillaries
Source: MRS Bull. 2024 Nov 19;50(1):44–51. doi: 10.1557/s43577-024-00813-7 (PMC11750922; doi:10.1557/s43577-024-00813-7)
Supplement: Supplementary file 1 — Supplementary file1 (PDF 169 KB) [file 43577_2024_813_MOESM1_ESM.pdf]

Filamentous cyanobacteria growth assessment using fluorinated ethylene propylene microcapillaries

David M.S. Silva[1], Raquel Amaral[1], Nuno M Reis[2], Paulo R.F. Rocha[1*]

[1] Bioelectronics & Bioenergy Research Lab, Centre for Functional Ecology (CFE), Department of Life Sciences, University of Coimbra, Coimbra, Portugal.

[2] Department of Chemical Engineering and Centre for Bioengineering and Biomedical Technologies (CBio), University of Bath, Claverton Down, Bath BA2 7AY, United Kingdom.

Contacts and Orcid:

David M.S. Silva, david.s.silva@uc.pt, 0009-0002-8701-9291

Raquel Amaral, r.amaral@uc.pt, 0000-0002-2197-6593

Nuno M Reis, nmr39@bath.ac.uk, 0000-0001-7500-8910

* Contact author Paulo R.F. Rocha, procha@uc.pt, 0000-0002-8917-9101

Supplementary Information

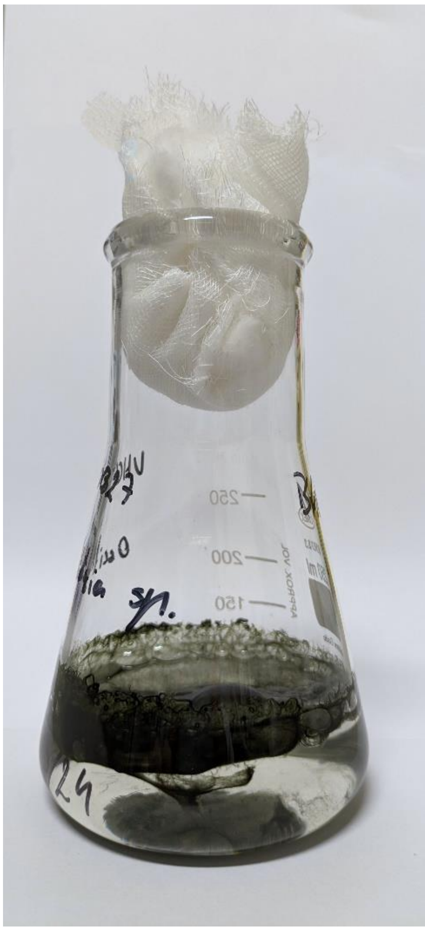

Online Resource 1 Culture of *Oscillatoria* sp. in Erlenmeyer flask
